# Supplementary material for: Non-Invasive versus Invasive Samples for Zika Virus Surveillance: A Comparative Study in New Caledonia and French Guiana in 2015–2016
Source: Microorganisms. 2021 Jun 16;9(6):1312. doi: 10.3390/microorganisms9061312 (PMC8235784; doi:10.3390/microorganisms9061312)
Supplement: Supplementary file 1 [file microorganisms-09-01312-s001.zip › microorganisms-1214746-supplementary.pdf]

Supplementary data

Table S1. Summary of symptoms and clinical signs related to patient samples.

Patient number	Days since onset of symptoms and clinical signs	Symptoms and clinical signs																		
		Arthralgia	Myalgia	Fever	Cephalalgia	Skin rash	Nausea	Retro-orbital pain	Diarrhea	Asthenia	Conjunctival hyperemia	Edema	Abdominal pain	Dysesthesia	Adenopathy	Cough	Hemoptoic sputum	Periorbital pain	Polyradiculoneuropathy	Thrombocytopenia
NC-1A	3				x	x				x					x					
NC-2A	4	Not specified																		
NC-3A	4	x	x	x		x														
NC-4A	3	x			x	x			x											
NC-5A	1	x	x		x		x	x		x										
NC-6A	4	x	x	x	x		x		x											
NC-7A	2		x	x	x					x										
NC-8A	3	x		x		x														
NC-9A	3	Not specified																		
NC-10A	5	x	x	x	x															
NC-11A	5	x	x	x		x														
NC-12A	1	x	x			x														
NC-13A	2	x	x	x	x		x													
NC-14A	2	x	x	x		x														
NC-15A	4	x	x	x	x	x								x						
NC-16A	5	x	x	x	x		x	x			x									
NC-17A	2	x	x		x	x	x													
NC-18A	2	x	x		x															
NC-19A	1	x	x	x	x	x	x													

NC-19B	2																			
NC-20A	6	x	x																	
NC-21A	1	x	x	x	x	x					x	x								
NC-22A	3	x	x	x	x															
NC-23A<br>NC-23B	2<br>6	x	x	x	x			x												
NC-24A	3		x	x	x															
NC-25A	4	x	x	x	x				x											
NC-26A<br>NC-26B	2<br>6	x	x	x	x	x					x	x								
NC-28A	7	Not specified																		
NC-29A	1	x	x	x	x															
NC-30A	4	Not specified																		
NC-31A	1	Not specified																		
NC-32A	1	x	x	x	x		x	x								x				
NC-33A	1	x	x	x	x			x												
NC-34A	2	x	x	x	x			x												
NC-35A	2	x	x	x	x		x	x	x											
NC-36A	3	x	x	x	x			x			x	x								
NC-37A<br>NC-37B	4<br>12	Not specified																		
NC-38A	2	x	x	x	x		x	x												
NC-39A	13	x	x	x	x			x												
NC-40A<br>NC-40B	7<br>25	x	x	x	x														x	
NC-41A<br>NC-41B	1<br>11	x	x	x	x		x	x												
NC-42A	2	x	x				x						x							
NC-43A	2	x	x	x	x															
NC-44A<br>NC-44B	5<br>7		x	x	x		x			x			x					x		x

NC-45A	3	x	x	x	x			x												
NC-46A<br>NC-46B	3<br>8	x		x			x	x	x					x						
NC-47A<br>NC-47B	3<br>11	x	x	x	x	x														
NC-48A	5	x		x	x		x	x					x				x			
GY-1A<br>GY-1B	2<br>3	x	x	x		x				x										
GY-4A<br>GY-4B	3<br>16	x	x	x		x					x									
GY-5A	4	x	x			x				x										
GY-6A<br>GY-6B	1<br>7	x																		
GY-7A	2					x														
GY-8A<br>GY-8B	6<br>9	x	x	x	x		x		x											

Table S2. Oligonucleotides used in the 4-plex RT-qPCR targeting both CHIKV and ZIKV [14].

Methods	Targeted genes	Oligonucleotides	Sequences	Concentration/reaction
CHIKV(a)	Nsp1	CHIKV(a)-Forward	TGATCCCGACTCAACCATCCT	600 nM
		CHIKV(a)-Reverse	GGCAAACGCAGTGGTACTTCCT	600 nM
		CHIKV(a)-Probe	6-FAM-TCCGACATCATCCTCCTTGCTGGC-BHQ-1	300 nM
CHIKV(b)	E1	CHIKV(b)-Forward	TCACTCCCTGTTGGACTTGATAGA	800 nM
		CHIKV(b)-Reverse	TTGACGAACAGAGTTAGGAACATACC	600 nM
		CHIKV(b)-Probe	HEX-AGGTACGCGCTTCAAGTTCGGCG-BHQ-1	400 nM
ZIKV(a)	M/A	ZIKV(a)-Forward	TTGGTCATGATACTGCTGATTGC	600 nM
		ZIKV(a)-Reverse	CCYTCCACAAAGTCCCTATTGC	600 nM
		ZIKV(1)-Probe	Texas Red-CGGCATACAGYATCAGGTGCATWGGAG-BHQ-2	300 nM
ZIKV(b)	E	ZIKV(b)-Forward	YCGYTGCCCAACACAAG	1000 nM
		ZIKV(b)-Reverse	CCACYAAYGTTCTTTTGCAGACAT	1000 nM
		ZIKV(b)-Probe	Cy5-AGCCTACCTTGACAAGCARTCAGACACTCAA-BHQ-2	500 nM

Table S3. Detection percentage of ZIKV in serum, urine and/or saliva samples according to days since onset of symptoms and clinical signs. The values are based on Table 1.

Days since onset of symptoms and clinical signs	Serum		Urine		Saliva		Urine or saliva	
	Total sample number	Positive samples (%)	Total sample number	Positive samples (%)	Total sample number	Positive samples (%)	Total sample number	Positive samples (%)
1	10	20	10	40	10	30	10	40
2	15	20	15	40	15	33.3	15	40
3	12	16.7	12	41.7	11	36.4	11	54.5
4	8	25	8	25	8	37.5	8	37.5
5	5	0	5	40	5	20	5	40
6	4	25	4	75	4	25	4	75
7	4	0	3	0	3	0	3	0
8	1	0	1	0	1	0	1	0
9	1	0	1	0	1	0	1	0
11	2	0	2	0	2	0	2	0
12	1	0	0	0	0	0	0	0
13	1	0	1	0	1	0	1	0
16	1	0	1	0	1	0	1	0
25	1	0	1	0	1	0	1	0
